# Metabolomics Fingerprint Predicts Risk of Death in Dilated Cardiomyopathy and Heart Failure

**DOI:** 10.3389/fcvm.2022.851905

**Published:** 2022-04-07

**Authors:** Alessia Vignoli, Alessandra Fornaro, Leonardo Tenori, Gabriele Castelli, Elisabetta Cecconi, Iacopo Olivotto, Niccolò Marchionni, Brunetto Alterini, Claudio Luchinat

**Affiliations:** ^1^Department of Chemistry “Ugo Schiff”, Magnetic Resonance Center (CERM), University of Florence, Sesto Fiorentino, Italy; ^2^Interuniversity Consortium for Magnetic Resonance of Metalloproteins, Sesto Fiorentino, Italy; ^3^Cardiomyopathy Unit, Careggi University Hospital, Florence, Italy; ^4^Division of Cardiovascular and Perioperative Medicine, Careggi University Hospital, Florence, Italy; ^5^Division of General Cardiology, Department of Experimental and Clinical Medicine, Careggi University Hospital, University of Florence, Florence, Italy

**Keywords:** metabolomics, heart failure, prognosis, NMR spectroscopy, precision medicine

## Abstract

**Background:**

Heart failure (HF) is a leading cause of morbidity and mortality worldwide. Metabolomics may help refine risk assessment and potentially guide HF management, but dedicated studies are few. This study aims at stratifying the long-term risk of death in a cohort of patients affected by HF due to dilated cardiomyopathy (DCM) using serum metabolomics *via* nuclear magnetic resonance (NMR) spectroscopy.

**Methods:**

A cohort of 106 patients with HF due to DCM, diagnosed and monitored between 1982 and 2011, were consecutively enrolled between 2010 and 2012, and a serum sample was collected from each participant. Each patient underwent half-yearly clinical assessments, and survival status at the last follow-up visit in 2019 was recorded. The NMR serum metabolomic profiles were retrospectively analyzed to evaluate the patient's risk of death. Overall, 26 patients died during the 8-years of the study.

**Results:**

The metabolomic fingerprint at enrollment was powerful in discriminating patients who died (HR 5.71, *p* = 0.00002), even when adjusted for potential covariates. The outcome prediction of metabolomics surpassed that of N-terminal pro b-type natriuretic peptide (NT-proBNP) (HR 2.97, *p* = 0.005). Metabolomic fingerprinting was able to sub-stratify the risk of death in patients with both preserved/mid-range and reduced ejection fraction [hazard ratio (HR) 3.46, *p* = 0.03; HR 6.01, *p* = 0.004, respectively]. Metabolomics and left ventricular ejection fraction (LVEF), combined in a score, proved to be synergistic in predicting survival (HR 8.09, *p* = 0.0000004).

**Conclusions:**

Metabolomic analysis *via* NMR enables fast and reproducible characterization of the serum metabolic fingerprint associated with poor prognosis in the HF setting. Our data suggest the importance of integrating several risk parameters to early identify HF patients at high-risk of poor outcomes.

## Introduction

Dilated cardiomyopathy (DCM), one of the leading causes of heart failure (HF) worldwide (1), is generally considered as a “final phenotype,” resulting from miscellaneous genomic or phenomic insults *via* activation of diverse DCM disease-causing cascades (2). The DCM may remain asymptomatic for years (3), eventually leading to progressive ventricular dilatation and both systolic and diastolic dysfunctions, arrhythmias, sudden death, and HF. The prevalence of DCM in observational studies of HF patients varied between 8 and 47%, while in trials of Heart Failure with Reduced Ejection Fraction (HFrEF), DCM etiology accounted for 12–35% of individuals (4).

Heart failure is a complex clinical syndrome in which heart function is inadequate to meet physiological demands and it constitutes a massive health problem, with a considerable residual disease burden due, at least in part, to a broad range of disease courses and responses to therapy (5). Several studies have aimed at defining the underlying pathophysiology of HF using recent advances in system biology approach as well as at discovering possible biomarkers to achieve a better prognostic stratification of cardiac failure (6, 7), beyond left ventricular ejection fraction (LVEF) evaluation. With this regard, several survival prediction models have been created using clinical risk scores and biomarkers, such as natriuretic peptides (8), but important knowledge gaps remain regarding the complex biological pathways determining individual variability. This limitation hinders the identification of specific patient subsets with different needs and fates.

In the era of precision medicine, Omics sciences could be the instrument to meet this need. Metabolomics is one of the latest omic technologies, broadly defined as the comprehensive measurement of the complete ensemble of endogenous and exogenous metabolites present in a biological specimen, which is the so-called metabolome (9). Metabolites represent, at the same time, the downstream output of the omics cascade, and the upstream input from various external factors, such as environment, lifestyle, diet, and drug administration (10). Thus, metabolites have been described as the most proximal reporters of any disease status or phenotype because their concentrations in biospecimens are directly related to the underlying pathophysiological landscape (11). Metabolomics applications in biomedical research are manifold (12–17), and this technology has already demonstrated its potentiality in the setting of cardiovascular diseases (18–23).

Metabolic impairment has long been identified as an intrinsic feature of HF pathophysiology and a detrimental self-perpetuating cycle involving heart failure and altered metabolism that promotes HF progression was postulated (24, 25). Energetic and structural metabolic failure is not limited to the myocardium, but it is reflected at a systemic level, and considerably contributes to major HF symptoms and disease progression (26, 27). Metabolomics has contributed to elucidating several systemic metabolic impairments that occur in patients with HF: insulin resistance, shift toward hyper-catabolism with blunting of anabolic pathways, impaired glucose oxidation and a switch toward glycolysis, impaired fatty acid β-oxidation, and urea cycle dysfunction (27–30). In our previous paper on the same cohort of patients, we showed that patients with HF were characterized by higher serum concentrations of phenylalanine, tyrosine, isoleucine, creatine, and low serum levels of lactate, citrate, lysine, and L-dopa (28). Moreover, metabolomics has been demonstrated to be a promising approach for the clinical prognosis of patients with HF (31–33).

Here, we propose a strategy for the prognostic evaluation of HF due to DCM based on the combination of serum nuclear magnetic resonance (NMR)-based metabolomics with traditional prognostic factors, such as N-terminal pro b-type natriuretic peptide (NT-proBNP) and LVEF. For this purpose, we retrospectively analyzed a well-defined, homogeneous, single-center cohort of patients with DCM with stable chronic HF diagnosed and monitored between 1982 and 2011 (median follow up time from DCM diagnosis: 15 years), and consecutively enrolled for this study between 2010 and 2012 (Supplementary Figure S1).

## Methods

### Patient Recruitment

**Figure 1:**
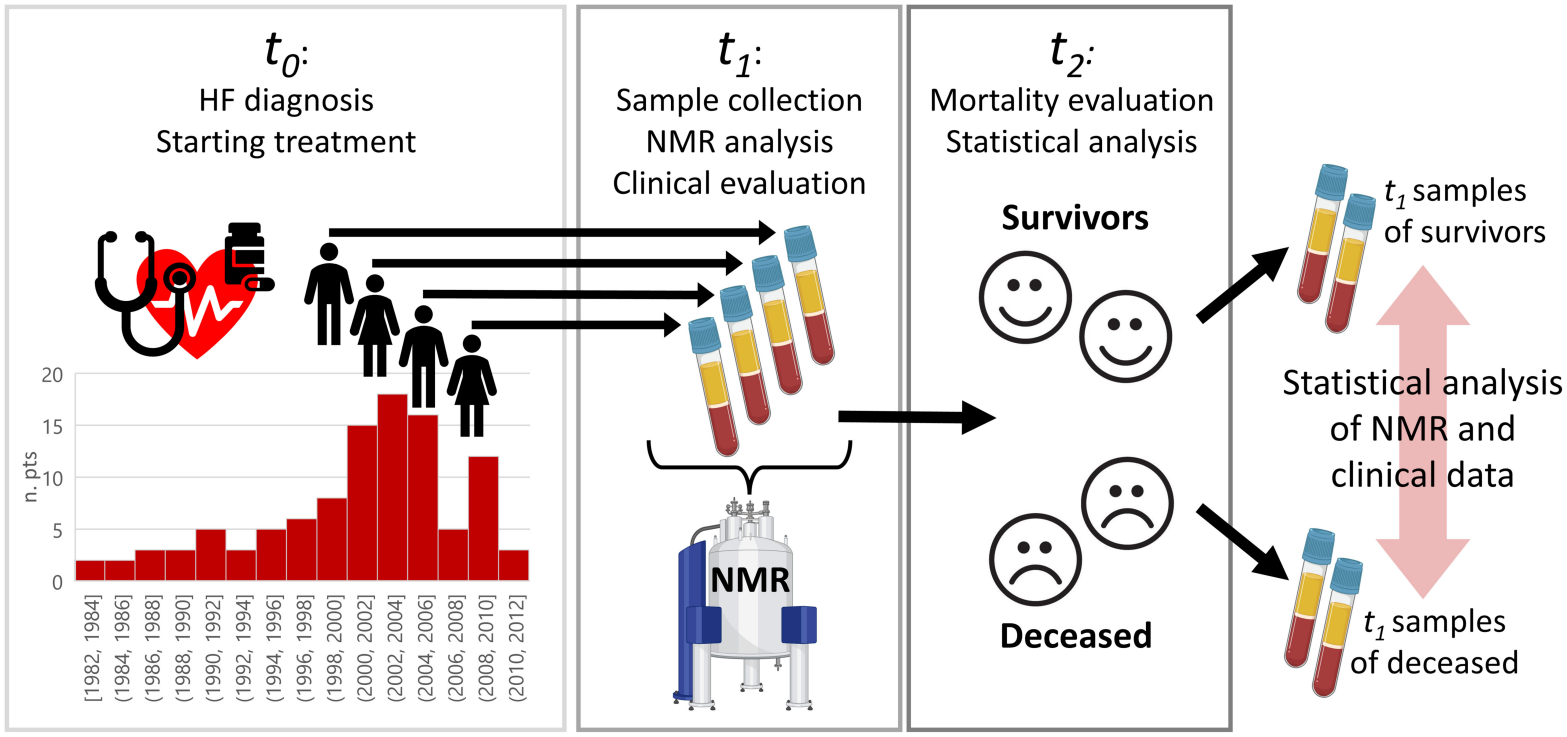
Graphical overview of the study design to investigate differences between survived and deceased patients with HF. Patients were diagnosed at time t0, where t0 for each patient was in the range 1982–2011. Between 2010 and 2012 (t1), the patients were re-examined or examined for the first time, blood serum samples collected and analyzed *via* nuclear magnetic resonance (NMR). Survival status was evaluated in 2019 (t2). Patients were, then, retrospectively split in two groups according to the survival status (alive vs. deceased), and statistical analysis on NMR data was performed.

In this retrospective study, a cohort of 106 adult patients (74 men, 32 women, median age 49, 95% CI 49–53 years) with chronic heart failure (i.e., at least one previous heart failure event, comprising hospitalization for HF and/or an urgent visit resulting in intravenous therapy for HF due to DCM) was examined. The present cohort is a sub-group of the population analyzed in our previous publication (28). The HF has been defined as a clinical syndrome characterized by fundamental symptoms (e.g., breathlessness, ankle swelling, and fatigue) and/or signs (e.g., elevated jugular venous pressure, pulmonary crackles, and peripheral edema) related to a structural and/or functional abnormality of the heart that results in elevated intracardiac pressures and/or inadequate cardiac output at rest and/or during exercise (34). A period of clinical stability of at least 6 months in optimal medical therapy (OMT) was required for enrollment. Patients were diagnosed between 1982 and 2011 (t0). In the period 2010–2012 (t1), patients were re-examined or examined for the first time (only for patients diagnosed between 2010 and 2012), blood serum samples were collected and analyzed *via* NMR. Then, survival status was evaluated at the last follow-up in 2019 (t2). In patients who died or were transplanted, the end of follow-up was considered either the time of death or heart transplantation. In the minority of patients lost to follow-up (i.e., not traceable by June 2019), the last clinical evaluation or the last telephone contact was considered. The experimental design is graphically illustrated in Figure 1.

The enrolled patients were classified as idiopathic DCM, defined by the presence of left ventricular (LV) or biventricular dilatation and systolic dysfunction in the absence of abnormal loading conditions (hypertension, valve disease) or coronary artery disease sufficient to cause global systolic impairment (1, 35). The patients with HF, judged to be secondary to ischemic heart disease, systemic hypertension, chemotherapy, alcoholic abuse, diabetes mellitus, *cor pulmonalis*, valve disease, or other cardiac or systemic diseases, were excluded, as well as patients for whom a coronary angiogram was not available. The patients were consecutively enrolled in the years 2010–2012 at the Careggi University Hospital Florence, Italy. They underwent half-yearly clinical assessments (median follow-up from enrollment 8 years). All study patients were evaluated and followed up by clinical history, physical examination, 12-lead ECG, standard chest radiograph, routine laboratory tests, M-mode, 2D, and Doppler echocardiography. For the entire study period, the patients were seen by the same cardiologists who assumed primary responsibility for their management.

### Ethical Issues

This study was approved by the local Ethics Committee (Azienda Ospedaliero—Universitaria Careggi, Florence, Italy). Written informed consent was obtained from each participant at the time of blood sample collection. The study adheres to the principles of the Helsinki Declaration and its later amendments.

### Collection of Samples

Each blood sample was collected through peripheral venous access in a 10-mL tube (BD P100, BD Diagnostics, Franklin Lakes, NJ). Subsequently, blood samples were centrifuged for 10 min at 4,000 rpm at the temperature of 4°C, then, the supernatant serum was aliquoted in sterile cryovials and stored at −80°C pending NMR analysis.

### NMR Analysis

Serum samples were prepared for NMR experiments as described in our previous publications (28, 36). One-dimensional ^1^H NMR spectra of each sample were acquired using a Bruker 600 MHz spectrometer (Bruker BioSpin) operating at 600.13 MHz proton Larmor frequency and equipped with a 5 mm CPTCI ^1^H-^13^C-^31^P and ^2^H-decoupling cryoprobe, including a z-axis gradient coil, an automatic tuning-matching, and an automatic sample changer. A BTO 2000 thermocouple served for temperature stabilization at the level of ~0.1 K at the sample. Before measurement, samples were kept for at least 3 min inside the NMR probe-head, for temperature equilibration at 310 K. For each serum sample, a standard nuclear Overhauser effect spectroscopy pulse sequence NOESY 1Dpresat (noesygppr1d.comp; Bruker BioSpin)(37), using 64 scans, 98,304 data points, a spectral width of 18,028 Hz, an acquisition time of 2.7 s, a relaxation delay of 4 s and a mixing time of 0.01 s (total duration of the NMR experiment 7 min), was applied to obtain a spectrum in which both signals of low molecular weight metabolites and high molecular weight macromolecules (i.e., proteins, lipids, and lipoproteins) are detected.

Free induction decays were multiplied by an exponential function equivalent to 1.0 Hz line-broadening factor before applying Fourier transform. The transformed spectra were automatically corrected for phase and baseline distortions and calibrated to the anomeric glucose doublet at δ 5.24 ppm using TopSpin3.6.2 (Bruker Biospinsrl).

Each 1D spectrum was segmented into 0.02 ppm chemical shift bins in the range 0.2–10 ppm, and the corresponding spectral areas were integrated using the AssureNMR software (Bruker BioSpin). The regions of residual water signal (4.37–5.13 ppm) and the signals of ethanol (1.12–1.23 ppm and 3.53–3.73 ppm) were removed, and the dimension of the system was reduced to 438 bins. The probabilistic quotient normalization (38) was applied on the remaining bins prior to statistical analysis.

### Statistical Analysis

Data analyses were performed using the open-source software R. Multivariate analysis was performed on binned spectra (thus, on the whole spectra, considering both assigned and unassigned metabolites). Principal component analysis (PCA) was used as the first unsupervised analysis to visualize data. Metabolomics analysis was performed using a fingerprinting approach: the metabolomic fingerprint is a global, rapid evaluation of an NMR spectrum as a whole that considers all (assigned or unassigned) detectable metabolites present in that biological sample (39). Standard partial least square discriminant analysis (40) (PLS-DA) was applied to discriminate the metabolomic fingerprints of survivors and deceased patients using the first 7 PLS components, and the PLS-DA model was validated using a Leave-One-Out cross-validation scheme (LOOCV, R script developed in-house). Since the group size is unbalanced (survived 75.5%, deceased 24.5%), samples from 25 survivors and 25 deceased patients were randomly chosen from the full dataset and subjected to PLS-DA modeling. The resampling procedure was performed 100 times to account for variability in the sampling procedure, and each model was cross-validated each time. Samples were assigned to one of the two classes using the majority vote algorithm (R library “mclust”) on the results obtained by the 100 iterations. Sensitivity, specificity, and accuracy were calculated according to the standard definitions. Variable importance in projections (VIP) was calculated using an R script developed in-house, variables with a VIP score higher than 1 were considered important in the PLS-DA model.

The predictive performance of the metabolomic PLS-DA classification was compared with that of LVEF and NT-proBNP. The LVEF classification followed the European Society of Cardiology guidelines (34), identifying three classes: reduced LVEF (<40%, high-risk: HiR), mid-range LVEF (40–49%, intermediate risk: IR), and preserved LVEF (≥50%, low risk: LR). Patients with a baseline level of NT-proBNP higher than 400 pg/ml were considered at high-risk of death. Moreover, the ability of the combination of metabolomics and LVEF in predicting poor prognosis was also tested. Metabolomics was used as the first screening method and then was adjusted using ejection fraction: patients predicted as survivors by the PLS-DA metabolomic model but with LVEF of <35% were reclassified as high-risk of death, while patients predicted as deceased but with LVEF of >50% were reclassified as low risk of death. All the above-mentioned analyses were performed using Kaplan–Meier (KM) curves, with the additional calculation of the hazard ratio (HR) and *p*-value assessed by the Log-Rank test (R library “survminer”). The performances and the independence of metabolomics were evaluated by calculating Cox proportional hazards regression models (41) (R library “Survival”) and each model significance was assessed through a likelihood-ratio test. These analyses were performed in a univariate and multivariate fashion.

The untargeted quantification of 22 metabolites and 114 lipoprotein-related parameters was performed using the Bruker IVDr analysis platform (42). The non-parametric Wilcoxon Rank-Sum test was used to infer differences between the groups of interest. The *P*-values were adjusted for multiple testing using the false discovery rate (FDR) procedure with Benjamini and Hochberg (43) correction at α = 0.05. Each metabolite/lipoprotein feature was divided into three tertiles and Cox regression models were calculated to estimate the association between metabolites/lipoproteins and prognosis. Additional models were calculated to adjust for additional covariates: sex, age at DCM diagnosis, time from DCM diagnosis and last follow up, NT-proBNP, LVEF, New York Heart Association (NYHA) class at enrollment, systolic blood pressure (SBP), end-diastolic diameter index (EDDi) at enrollment, and left atrial volume index (LAVi) at enrollment.

Robust correlations were calculated among metabolomic variables and clinical data following the 10% winsorized correlation approach (44) using the function “wincor” of the R package “WRC2.” The *P*-values were adjusted for multiple testing using the FDR procedure.

## Results

### Study Population

Baseline characteristics of the cohort are shown in Table 1. The median age at enrollment was 58.5 ± 14.1 years, with a median age at DCM diagnosis of 49 ± 11.9 years. The patients were predominantly men (69.8%). The median time from DCM diagnosis at last evaluation was 15 ± 5.9 years. The patients were mostly pauci-symptomatic at enrollment (85.8% NYHA class I-II). Median systolic blood pressure (SPB) was 120 ± 14.8 mmHg, while echocardiographic parameters showed a considerable LV (left ventricular) and LA (left atrial) enlargement in most patients, with median indexed LVEDDi and LAVi of 31.6 ± 4.9 and 41.9 ± 18 mm/m^2^, respectively. Median LVEF at enrollment was 44.5 ± 8.15% with an NT-proBNP median value of 219.1 ± 237.8 pg/ml. All patients were on ACE-inhibitors or angiotensin receptor blockers inhibitors (ARBs) at enrollment, while 92.4% were receiving beta-blockers (BB). The introduction of Angiotensin Receptor Neprilysin Inhibitors (ARNIs) into clinical practice has become significant only in the last 2 years of the study follow-up, therefore, this agent has not been taken into consideration. Diuretic treatment was administered in 71% of patients at baseline.

**Table 1:** Baseline characteristics of enrolled patients.

	**Overall at t_**1**_**	**Alive at t_**2**_**	**Deceased at t_**2**_**	***p*-value^#^**
	**(106 pts)**	**(80 pts)**	**(26 pts)**	
Age at enrollment (yrs), median (mad)	58.5 (14.1)	56.0 (11.9)	67.5 (12.6)	0.004
Gender (M), *n* (%)	74 (69.8)	55 (68.8)	19 (73.1)	0.9
Age at DCM diagnosis (yrs), median (mad)	49 (11.9)	48.5 (11.1)	55.5 (17.0)	0.1
Time from DCM diagnosis and last follow up (yrs), median (mad)	15 (5.9)	15 (5.9)	15 (7.4)	0.9
NYHA class at enrollment				0.07
I, *n* (%)	38 (35.8)	31 (38.8)	7 (26.9)	
II, *n* (%)	53 (50.0)	41 (51.2)	12 (46.2)	
III, *n* (%)	13 (12.2)	8 (10.0)	5 (19.2)	
IV, *n* (%)	2 (2.0)	0 (0.0)	2 (7.7)	
SBP (mmHg) at enrollment, median (mad)	120 (14.8)	120 (14.8)	110 (14.8)	0.02
EDDi (mm/mq) at enrollment, median (mad)	31.6 (4.9)	30.8 (3.8)	36.9 (5.7)	0.004
LAVi (mL/mq) at enrollment, median (mad)	41.9 (18.0)	41.3 (16.3)	47.8 (26.9)	0.09
LVEF (%) at enrollment, median (mad)	44.5 (8.15)	46.0 (7.4)	37.5 (9.6)	0.001
NT-proBNP (pg/ml) at enrollment, median (mad)	219.1 (237.8)	180.6 (176.0)	613.3 (741.9)	0.005
BB^*^ at enrollment, *n* (%)	98 (92.4)	73 (91.2)	25 (96.1)	0.8
ACE-I^§^ at enrollment, *n* (%)	106 (100)	80 (100)	26 (100)	1
Diuretic at enrollment, *n* (%)	75 (70.7)	51 (63.8)	24 (92.3)	0.02

*DCM, dilated cardiomyopathy; NYHA, New York Heart Association Classification; SBP, systolic blood pressure; EDDi, indexed end diastolic diameter; LAVi, indexed left atrial volume; LVEF, left ventricular ejection fraction; NT-proBNP, N-terminal pro b-type natriuretic peptide; BB, beta blockers; ACE-I, Angiotensin-converting-enzyme inhibitors*.

* *bisoprolol-equivalent*;

§ *ramipril-equivalent*;

# *FDR adjusted with the Benjamini-Hochberg procedure*.

*Data are reported for the overall population at enrollment (t1) and for alive and deceased patients at follow-up separated (t2)*.

At the last evaluation, 80 patients were alive, while 26 had died for HF-related causes (7 patients for sudden cardiac death and 19 patients for refractory heart failure). Demographic and clinical characteristics of the 106 patients with DCM depending on survival status at t2 are shown in Table 1: age, SBP, EDDi, LVEF, NT-proBNP, and diuretic administration at enrollment were significantly different between survivors and deceased patients.

### NMR Metabolomics Prediction of HF Prognosis

**Figure 2:**
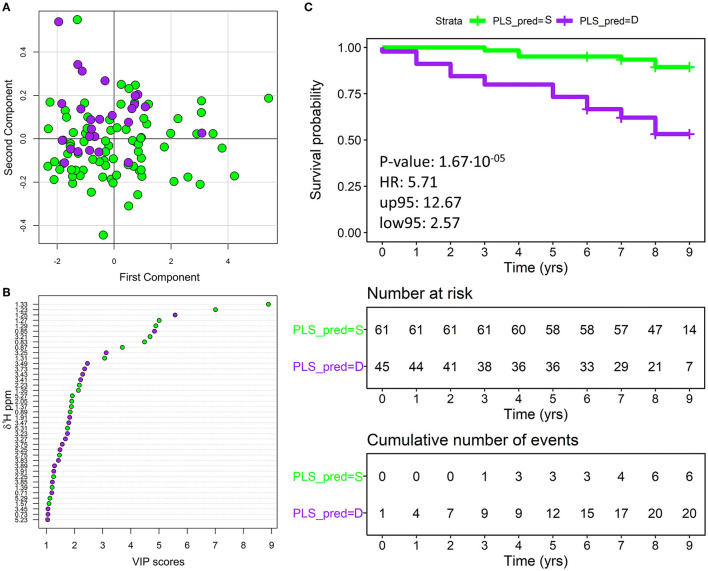
**(A)** score plot of the first two components of the partial least square discriminant analysis (PLS-DA) model discriminating survivor (80 green dots) and deceased (26 purple dots) patients. **(B)** The variable importance in projections (VIP) score plot indicating the most discriminating bins in descending order of importance; only the bins that showed a VIP score>1 are reported in the plot. For each bin the starting ppm is reported. Green dots represent bins with intensity higher in survivor patients, conversely purple dots represent bins with intensity higher in deceased patients. **(C)** Overall patients with HF, plotting actual survival over time (in years) according to estimated metabolomic risk [Kaplan-Meier (KM) curves]. Low metabolomic risk (green) and high metabolomic risk (purple) patients are significantly clustered with a *p*-value of 1.67·10^−5^ (calculated with the Log-Rank test) and an HR of 5.71. Censored events represent either the time of last recorded clinical follow-up, or the time of death. Number at risk: number of patients stratified according to the metabolomics classification at each time point. Cumulative number of events: total number of deceased patients at each time point for each metabolomics risk group. PLS_PRED = S, predicted by PLS as survivor; PLS_PRED = D, predicted by PLS as deceased.

Before any supervised approach, PCA was used as an exploratory analysis to visualize data. No outlier or relevant clustering emerged from the score plot (Supplementary Figure S2). Then, the differences in serum metabolomics ^1^H NMR fingerprints of survivors and deceased patients were analyzed using standard PLS-DA (Figure 2A). To ensure that the calculated PLS-DA model was statistically robust, an internal validation using a LOOCV was performed: the two groups show a good clustering, yielding 70.8% accuracy, 76.9% sensitivity, and 68.8% specificity (deceased patients wrongly classified as survivors present, on average, a longer survival time between enrollment and death: 5.7 vs. 4.3 years, see Supplementary Figure S1). Based on the VIP score analysis (Figure 2B) variables that mainly contributed to the PLS-DA model, and, thus, to the risk of stratification, were related to spectral regions of creatine, creatinine, lactate, trimethylamine-N-oxide, and lipoproteins.

Analyzing the metabolomic classification with KM curves (Figure 2C), clear discrimination resulted between patients who died and those who survived: *p*-value of 0.00002 and HR of 5.71 (95% CI 2.57–12.67). The use of the majority vote algorithm for patient classification produced a gray region in which patients were assigned to a class or another with a small margin (50 ± 10%). To verify the robustness of our model, we repeated the KM analyses after removing the 11 ambiguous patients that were classified with a margin < 10%. Removing borderline patients did not significantly affect the overall model accuracy (Supplementary Figure S3).

### Comparison of Metabolomics With Known Prognostic Factors: NT-ProBNP and Ejection Fraction

The prognostic significance of NT-proBNP natriuretic peptide, examined at enrollment, was analyzed. The results of the KM analyses are shown in Supplementary Figure S4. Compared to metabolomics results, NT-proBNP showed higher specificity (71.2%) in discriminating deceased and survivor patients, although with lower sensitivity (58.3%) and accuracy (68%). The combination of NT-proBNP and metabolomics provided only a slight improvement in the outcome prediction: 75% sensitivity, 68.5% specificity, and 70.1% accuracy.

**Figure 3:**
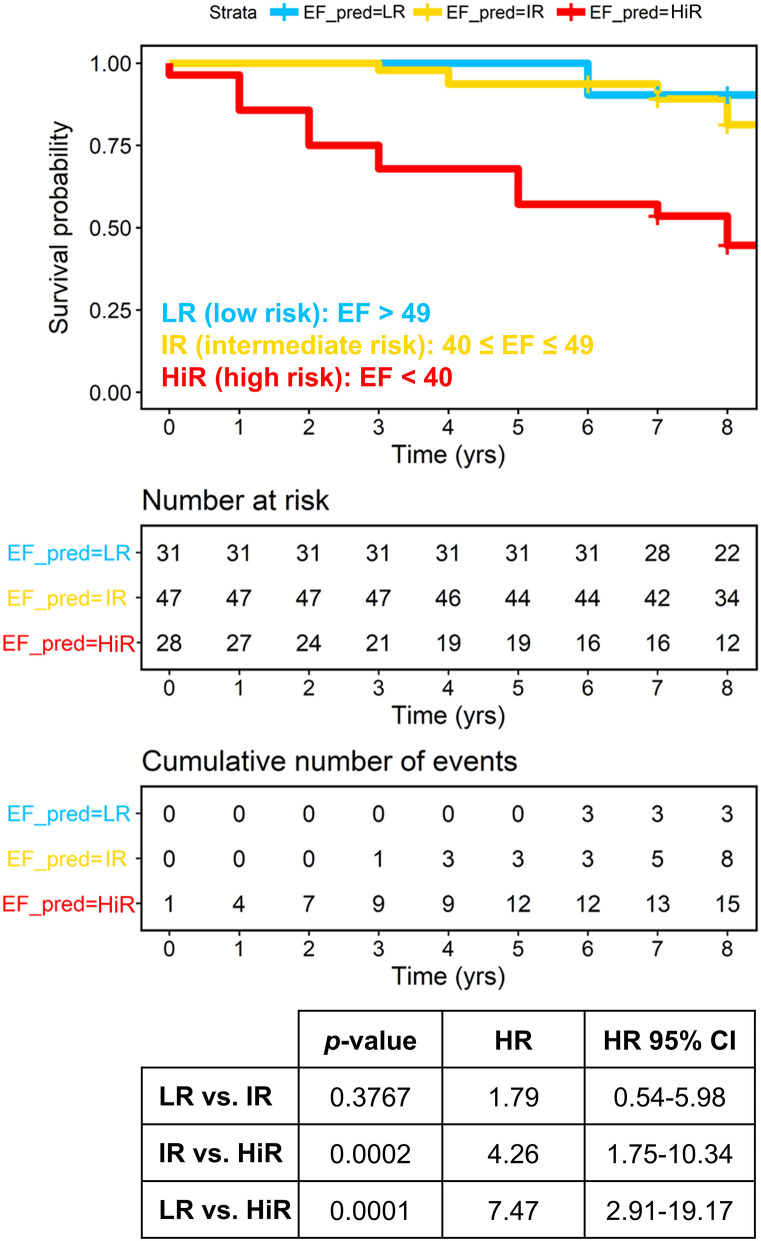
Overall patients with HF, plotting actual survival over time (measured in years) according to risk estimated based on ejection fraction (Kaplan-Meier curves). High-risk (red) group is significantly clustered with respect to both intermediate (yellow) and low (blue) risk groups with HR of 4.26 and 7.47, respectively. Censored events represent either the time of last recorded clinical follow up, or time of death. Number at risk: number of patients stratified according to the left ventricular ejection fraction (LVEF) at each time point. Cumulative number of events: total number of deceased patients at each timepoint for each risk group based on the LVEF. The *P*-values are calculated with the Log-Rank test. EF_PRED = LR: predicted by EF at low risk of death; EF_PRED = IR: predicted by EF at intermediate risk; EF_PRED = HR: predicted by EF at high-risk.

The predictive performance of the metabolomic model was also compared with that of left ventricular ejection fraction, and the results are reported in Figure 3. Patients with reduced LVEF showed a significantly higher risk of death (HRs of 7.47 and 4.26 and *p*-values of 0.0001 and 0.0002 concerning LR and IR, respectively), whereas patients with mid-range and preserved LVEF were both associated with a lower risk (HR 1.79, *p*-value 0.38).

**Figure 4:**
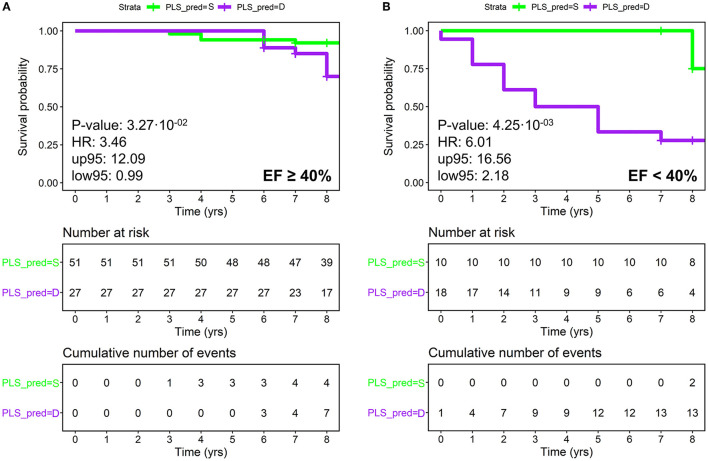
Patients with HF divided according to LVEF subclasses **(A)** LVEF ≥ 40% (low risk), **(B)** LVEF < 40% (high-risk), plotting actual survival over time (measured in years) according to estimated risk based on metabolomics (KM curves). Low-risk patients are colored in green and high-risk in purple. The *P*-values are calculated using the Log-Rank test. Censored events represent either the time of last recorded clinical follow-up, or time of death. Number at risk: number of patients stratified according to metabolomics. Cumulative number of events: total number of deceased patients at each time point for each metabolomic group. **(A)** KM analysis on metabolomic classification of patients with LVEF ≥ 40%; **(B)** KM analysis on metabolomic classification of patients with LVEF < 40%. PLS_PRED = S, predicted by PLS as survivor; PLS_PRED = D, predicted by PLS as deceased.

The potential of metabolomics in sub-stratifying LVEF classes was then tested: mid-range and preserved LVEF were considered as a single low-risk class, whereas reduced LVEF was considered as a high-risk class. Although metabolomics showed to effectively sub-stratify low- and high-risk patients in both classes (HRs 3.46 and 6.01, *p*-values 0.03 and 0.004, respectively), it showed the best performance in the high-risk class (Figure 4).

**Figure 5:**
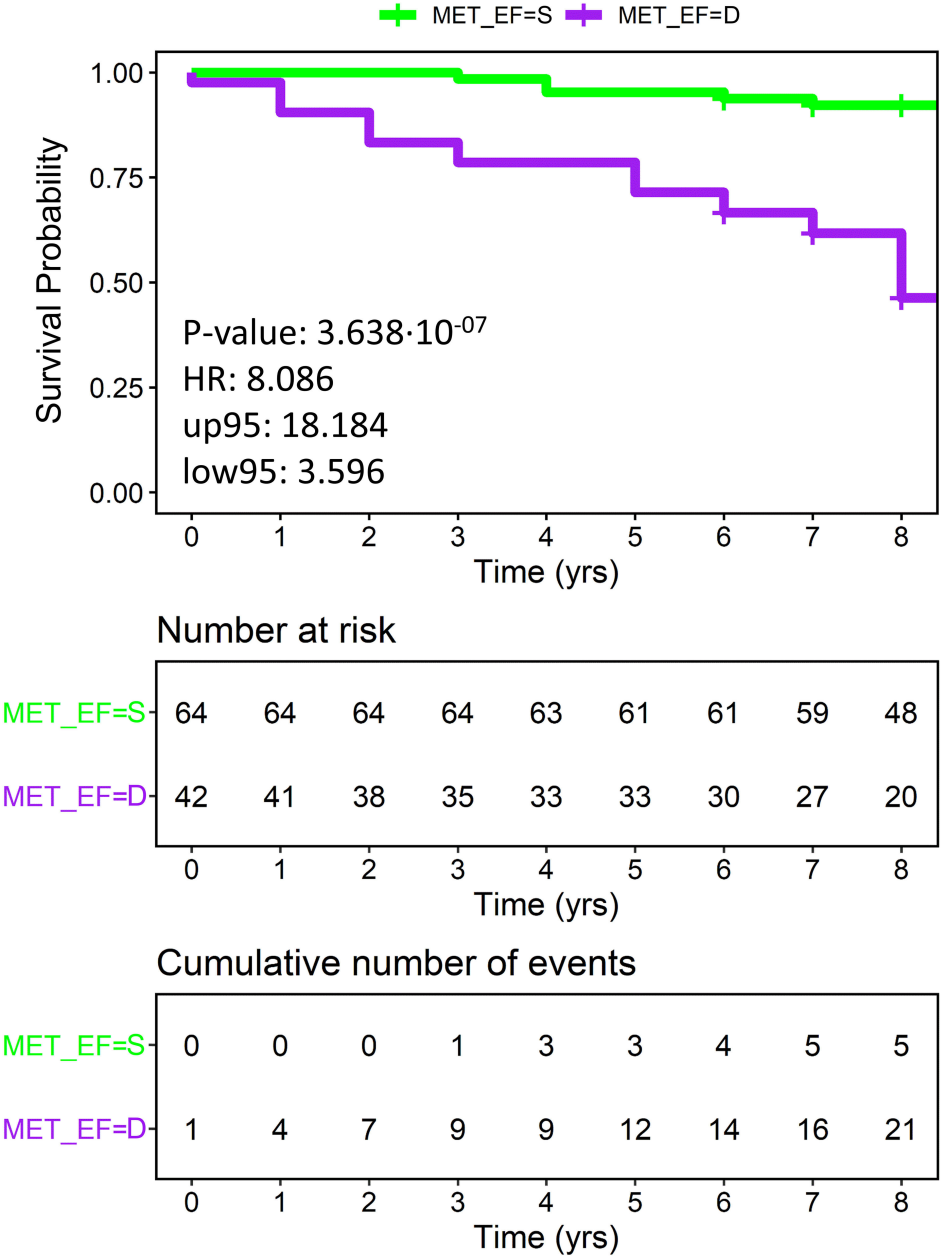
Overall patients with HF, plotting actual survival over time (measured in years) according to risk estimated using a combination of metabolomics and ejection fraction (KM curves). Low metabolomic risk (green) and high metabolomic risk (purple) patients are significantly clustered with a *p*-value of 3.64·10^−7^ (calculated with the Log-Rank test) and an HR of 8.09. Censored events represent either the time of last recorded clinical follow-up, or the time of death. Number at risk: number of patients stratified according to the combined score at each time point. Cumulative number of events: total number of deceased patients at each time point for each risk group based on the combined score. MET_EF = S, predicted by the combined score of metabolomics and EF as survivor; MET_EF = D, predicted by the combined score of metabolomics and EF as deceased.

Finally, the outcome prediction of the combination of metabolomics and LVEF was tested. The resulting combined score relies on LVEF for <35 or >50% values (i.e., when LVEF appears highly prognostic), whilst it is based on metabolomics for intermediate LVEF values, for which LVEF shows the least predictive capacity. This combined approach led to an improved prediction, with 75.5% accuracy, 73.8% sensitivity, 80.8% specificity, HR 8.09, and *p*-value 0.0000004 as shown by KM analysis (Figure 5).

### Analysis of Clinical Covariates

Possible covariates, such as advanced age at diagnosis (>60 years), sex, the time elapsed since the first diagnosis, NYHA functional class, SBP and echocardiographic parameters (EDDi and LAVi), were compared with the three main prognostic parameters (metabolomics, NT-proBNP, and LVEF) *via* univariate and multivariate Cox regression analyses (results displayed in Table 2). After univariate analysis, metabolomics, age at diagnosis, advanced NYHA classes (III-IV) at enrollment, NT-proBNP, and reduced LVEF resulted to be statistically associated with the outcome; however, after multivariate Cox regression model, only metabolomics (HR 6.72, *p* 0.0007), advanced age at diagnosis (HR 3.26, *p* 0.008), and reduced LVEF (HR 2.6, *p* 0.001) maintained their correlation with prognosis, regardless of other variables.

**Table 2:** Association with the outcome: unadjusted and adjusted hazard ratios (HR).

	**Hazard ratio**	***p*-value**	**Hazard ratio**	***p*-value**
	**(univariate)**		**(multivariate)**	
**Metabolomics**				
High-risk	5.92 (2.37–14.76)	<0.001	7.00 (2.28–21.51)	<0.001
**Sex**				
Male	1.18 (0.49–2.80)	0.72	0.64 (0.24–1.73)	0.38
**Age at DCM diagnosis**				
>60 yrs	2.22 (1.01–4.90)	0.048	4.40 (1.64–11.79)	<0.01
**Time from DCM diagnosis and last follow up**				
>15 yrs	1.02 (0.47–2.20)	0.96	0.91 (0.36–2.31)	0.84
**NT-proBNP**				
>400 pg/mL	3.03 (1.34–6.82)	<0.01	1.30 (0.43–3.94)	0.64
**LVEF**				
40 ≤ EF ≤ 49 (IR)	1.80 (0.48–6.78)	0.39	1.80 (0.41–7.94)	0.44
<40 (HiR)	8.09 (2.34–27.99)	<0.001	9.35 (1.81–48.26)	<0.01
**NYHA class at enrollment**				
II	1.24 (0.49–3.14)	0.65	0.48(0.12–2.02)	0.32
III–IV	3.52 (1.23–10.06)	0.019	0.66(0.12–3.61)	0.63
**SBP**				
>130 mmHg	0.80 (0.28–2.33)	0.68	0.85 (0.26–2.72)	0.78
**EDDi at enrollment**				
>30 mm/mq	1.72 (0.69–4.28)	0.24	0.89 (0.28–2.84)	0.85
**LAVi at enrollment**				
>40 mL/mq	1.76 (0.77–4.06)	0.18	0.60 (0.18–1.98)	0.40

*Correlation with the outcome for prognostic features and metabolomics score using univariate and multivariate Cox regression analysis. In the multivariate hazard ratios of all the variables were included together in the analysis*.

*DCM, dilated cardiomyopathy; NYHA, New York Heart Association Classification; SBP, systolic blood pressure; EDDi, indexed end diastolic diameter; LAVi, indexed left atrial volume; LVEF, left ventricular ejection fraction; NT-proBNP, N-terminal pro b-type natriuretic peptide*.

Correlations among metabolomics (metabolites and lipoprotein main parameters) and clinical variables are shown in Supplementary Figure S5. Several statistically significant correlations emerged from this analysis. In particular, succinic acid, acetone, and trimethylamine-N-oxide show a strong correlation pattern with NT-proBNP and with several echocardiographic (ECO) parameters (ECO left the atrial end-diastolic area, ECO left atrial end-diastolic diameter, ECO left atrial end-diastolic, and systolic volume). Also, glucose, lactic acid, and citric acid present correlations with the abovementioned echocardiographic parameters. Glycated hemoglobin (Hb1AC) significantly correlates with 12 metabolites. The LVEF anticorrelates with trimethylamine-N-oxide and correlates with creatine, Apo-A1, and Apo-2. Trimethylamine-N-oxide and creatine show the same trend of correlation with systolic blood pressure. Estimated glomerular filtration rate anticorrelates with creatinine and correlates with high-density lipo-protein (HDL) cholesterol, body mass index (BMI) correlates with phenylalanine and tyrosine, and electrocardiogram QT interval shows positive correlations with valine, pyruvic and citric acids.

Several statistically significant correlations also emerged among quantified metabolites and many lipoprotein-related parameters. In particular, amino acids, as well as lipoproteins, are shown to be highly intercorrelated. All results are presented in Supplementary Figure S6.

### Association Between Metabolic Features and Prognosis

Univariate analysis of the quantified metabolomic features (Supplementary Table S1), using the Wilcoxon test, unraveled that deceased patients as compared with survivors were characterized at enrollment by lower levels of creatine, apolipoprotein (apo-)A2 HDL, apo-A2, phospholipids HDL-3, apo-A1 HDL-3, apo-A1 HDL-4, apo-A2 HDL-4, phospholipids VLDL, phospholipids HDL-4, apo-A2 HDL-3, free cholesterol VLDL-3, triglycerides HDL-4, cholesterol HDL-3, and by higher levels of trimethylamine-N-oxide, creatinine, lactate, LDL and HDL Cholesterol ratio, triglycerides LDL-3, and triglycerides LDL-2 (*p*-value <0.05 for all before FDR correction).

Concentrations of each metabolite/lipoprotein were used to build Cox regression models for the evaluation of their net effect on survived and deceased patients (Table 3). Multivariate models adjusted for sex, age at DCM diagnosis, time from DCM diagnosis and last follow up, NT-proBNP, LVEF, NYHA class at enrollment, SBP, EDDi at enrollment, and LAVi at enrollment were also calculated. In the univariate model, higher levels of trimethylamine-N-oxide (3rd tertile), creatinine (3rd tertile), acetic acid (2nd tertile), succinic acid (3rd tertile), triglycerides low-density lipoprotein (LDL) (2nd tertile), and triglycerides LDL-3 (3rd tertile) are associated with a higher risk of poor outcome, whereas higher levels of triglycerides very-low-density lipoprotein (VLDL) (3rd tertile), Apo-A2 HDL (2nd and 3rd tertiles), triglycerides, VLDL-3 (3rd tertile), phospholipids (VLDL-1 (3rd tertile), phospholipids VLDL-3 (3rd tertile), and triglycerides HDL-4 (3rd tertile) are associated with a good prognosis. Among them, only the associations related to trimethylamine-N-oxide (3rd tertile), triglycerides LDL (2nd tertile), phospholipids VLDL-3 (3rd tertile), triglycerides LDL-3 (3rd tertile), and triglycerides HDL-4 (3rd tertile) remain statistically significant in the multivariate model adjusted for clinical covariates. However, in addition to these variables, additional ones (that were not significant in the univariate model) related to various LDL subfractions 1, cholesterol HDL-2, and Apo-B100 Apo-A1 ratio resulted to be significant in the multivariate model (Table 3).

**Table 3:** Association between metabolites/lipoproteins and the outcome: results of univariate and multivariate (adjusted for sex, age at DCM diagnosis, time from DCM diagnosis and last follow up, NT-proBNP, LVEF, NYHA class at enrollment, SBP, EDDi at enrollment, LAVi at enrollment) Cox regression analyses are reported.

	**Hazard ratio**	***p*-value**	**Hazard ratio**	***p*-value**
	**(univariate)**		**(multivariate)**	
Trimethylamine-N-oxide (2nd tertile)	3.33	0.0678	7.34	0.0148
Trimethylamine-N-oxide (3rd tertile)	5.94	0.0054	4.69	0.0323
Creatinine (2nd tertile)	1.28	0.6565	0.87	0.8352
Creatinine (3rd tertile)	2.69	0.0453	1.11	0.8673
Acetic acid (2nd tertile)	3.25	0.0414	2.39	0.1646
Acetic acid (3rd tertile)	2.90	0.0718	3.68	0.0534
Succinic acid (2nd tertile)	1.17	0.7895	0.67	0.5605
Succinic acid (3rd tertile)	4.24	0.0008	2.32	0.2101
Pyruvic acid (2nd tertile)	0.38	0.1000	0.09	0.0012
Pyruvic acid (3rd tertile)	1.24	0.6193	0.92	0.8852
Calculated Figures, Apo-B100/Apo-A1 (2nd tertile)	1.70	0.3048	3.26	0.0415
Calculated Figures, Apo-B100/Apo-A1 (3rd tertile)	1.97	0.1880	2.27	0.1732
Calculated Figures, LDL-1 Particle Number (2nd tertile)	1.21	0.7176	3.59	0.0494
Calculated Figures, LDL-1 Particle Number (3rd tertile)	1.78	0.2336	4.10	0.0192
Lipoprotein Main Fractions, Triglycerides, VLDL (2nd tertile)	0.98	0.9544	0.86	0.7625
Lipoprotein Main Fractions, Triglycerides, VLDL (3rd tertile)	0.32	0.0457	0.47	0.2199
Lipoprotein Main Fractions, Triglycerides, LDL (2nd tertile)	4.23	0.0110	4.02	0.0262
Lipoprotein Main Fractions, Triglycerides, LDL (3rd tertile)	2.30	0.1728	2.57	0.1579
Lipoprotein Main Fractions, Phospholipids, IDL (2nd tertile)	0.44	0.0967	0.42	0.1058
Lipoprotein Main Fractions, Phospholipids, IDL (3rd tertile)	0.53	0.1804	0.80	0.6833
Lipoprotein Main Fractions, Phospholipids, LDL (2nd tertile)	1.82	0.2466	3.22	0.0399
Lipoprotein Main Fractions, Phospholipids, LDL (3rd tertile)	1.77	0.2703	1.99	0.2403
Lipoprotein Main Fractions, Apo-A2, HDL (2nd tertile)	0.33	0.0222	0.41	0.1127
Lipoprotein Main Fractions, Apo-A2, HDL (3rd tertile)	0.28	0.0140	0.45	0.1562
VLDL Subfractions, Triglycerides, VLDL-3 (2nd tertile)	0.96	0.9173	0.93	0.8845
VLDL Subfractions, Triglycerides, VLDL-3 (3rd tertile)	0.24	0.0290	0.22	0.0573
VLDL Subfractions, Phospholipids, VLDL-1 (2nd tertile)	0.94	0.8898	0.48	0.1503
VLDL Subfractions, Phospholipids, VLDL-1 (3rd tertile)	0.32	0.0462	0.41	0.1622
VLDL Subfractions, Phospholipids, VLDL-3 (2nd tertile)	0.71	0.4346	0.84	0.7121
VLDL Subfractions, Phospholipids, VLDL-3 (3rd tertile)	0.30	0.0353	0.24	0.0358
LDL Subfractions, Triglycerides, LDL-1 (2nd tertile)	2.23	0.1435	3.44	0.0620
LDL Subfractions, Triglycerides, LDL-1 (3rd tertile)	2.65	0.0713	4.25	0.0243
LDL Subfractions, Triglycerides, LDL-3 (2nd tertile)	1.04	0.9447	0.55	0.3732
LDL Subfractions, Triglycerides, LDL-3 (3rd tertile)	2.88	0.0302	3.77	0.0418
LDL Subfractions, Triglycerides, LDL-6 (2nd tertile)	0.67	0.3796	0.32	0.0389
LDL Subfractions, Triglycerides, LDL-6 (3rd tertile)	0.49	0.1568	0.36	0.0859
LDL Subfractions, Cholesterol, LDL-1 (2nd tertile)	1.39	0.5176	2.95	0.0739
LDL Subfractions, Cholesterol, LDL-1 (3rd tertile)	1.55	0.3720	4.58	0.0161
LDL Subfractions, Cholesterol, LDL-2 (2nd tertile)	1.98	0.2112	2.15	0.2501
LDL Subfractions, Cholesterol, LDL-2 (3rd tertile)	2.23	0.1379	4.21	0.0287
LDL Subfractions, Free Cholesterol, LDL-1 (2nd tertile)	1.39	0.5176	2.84	0.1002
LDL Subfractions, Free Cholesterol, LDL-1 (3rd tertile)	1.55	0.3720	4.29	0.0251
LDL Subfractions, Free Cholesterol, LDL-2 (2nd tertile)	1.80	0.2938	3.55	0.0854
LDL Subfractions, Free Cholesterol, LDL-2 (3rd tertile)	2.43	0.0951	5.13	0.0211
LDL Subfractions, Phospholipids, LDL-1 (2nd tertile)	1.41	0.4965	3.47	0.0401
LDL Subfractions, Phospholipids, LDL-1 (3rd tertile)	1.60	0.3427	3.66	0.0460
LDL Subfractions, Phospholipids, LDL-2 (2nd tertile)	2.02	0.1998	2.05	0.2744
LDL Subfractions, Phospholipids, LDL-2 (3rd tertile)	2.25	0.1336	3.96	0.0374
LDL Subfractions, Apo-B, LDL-1 (2nd tertile)	1.21	0.7176	3.59	0.0494
LDL Subfractions, Apo-B, LDL-1 (3rd tertile)	1.78	0.2336	4.10	0.0192
HDL Subfractions, Triglycerides, HDL-4 (2nd tertile)	0.64	0.2996	0.45	0.1045
HDL Subfractions, Triglycerides, HDL-4 (3rd tertile)	0.28	0.0276	0.25	0.0433
HDL Subfractions, Cholesterol, HDL-2 (2nd tertile)	0.42	0.0830	0.26	0.0391
HDL Subfractions, Cholesterol, HDL-2 (3rd tertile)	0.62	0.2910	0.46	0.1834

*For each variable the reference is the first tertile group*.

## Discussion

Heart failure is a complex syndrome and constitutes the ultimate result of several cardiovascular injuries. The DCM is one of the most frequent causes of HF and heart transplantation, and in turn, constitutes the final phenotype derived from multiple pathophysiological mechanisms that mainly involve the structure and the energetic metabolism of cardiomyocytes. Prognostic evaluation of patients with DCM, particularly when complicated by HF, is a crucial point in the clinical process. Among traditional risk factors, LVEF still represents the cornerstone of prognostic stratification and has an essential role in phenotyping and guiding the therapy of patients with chronic HF (45), albeit with limitations related to the operator-dependent variability and scarce precocity. As regards to natriuretic peptides, there is a considerable experience both in DCM and in HF (46): BNP or NT-proBNP levels increase the accuracy of diagnosis of HF in the emergency department (47), as well as the prognosis at the time of hospital discharge (48).

Depending on LVEF, HF is currently classified into three subgroups: reduced (HFrEF), mildly-reduced (HFmrEF), and preserved ejection fraction (HFpEF) (34). The prognosis of HF subtypes appears to be similar, although patients with HFmrEF have higher readmission rates than patients with HFpEF, they share comparable mortality rates with patients with HFrEF and patients with HFpEF (49), even though ambulatory patients with HFmrEF show lower mortality than those with HFrEF, more akin to those with HFpEF. Moreover, patients with HFmrEF may include patients whose LVEF is increased from ≤40% or declined from ≥50% (50). Nonetheless, HF spans the entire range of LVEF (as an abnormally distributed variable), and measurement by echocardiography is subject to substantial variability, making the complexity of the phenotypes as well as their prognosis and management even more tricky. The level of neurohumoral activity and the response to medical therapies change among HF subtypes, suggesting differences in their underlying pathophysiology (51), which could be captured by the multiformity of the metabolomic fingerprints. Indeed, the metabolome represents what is happening in the body, providing the analysis of patients and their biological idiosyncrasies within the dynamic context of a disease process like HF.

Given these premises, our study aimed to evaluate the prognostic power of NMR metabolomic fingerprinting in predicting survival in a well-defined cohort of oligosymptomatic patients with HF with DCM over a median follow-up of 8 years. In our cohort, the overall mortality was 25%. Patients who deceased at baseline were older and showed larger LV, with lower LVEF and SBP values. Even if not reaching statistical significance, they tended to be more symptomatic and with larger LAVI. The NT-proBNP levels were significantly higher in patients who died. There were no differences in HF treatment among deceased patients concerning survivors. The NMR fingerprinting well-discriminated the survived and deceased patients with 70.8% accuracy (HR 5.71, *p* < 0.0001), and the metabolomic parameters that mainly contributed to the discrimination were trimethylamine-N-oxide, creatine, creatinine, lactate, and several lipoprotein-related parameters (Table 3; Supplementary Table S2).

Energetic metabolism plays a pivotal role in the onset and evolution of HF (52). Creatine is a key player in sustaining energy metabolism and functions of tissues with high energy demand, such as the myocardium (53). Indeed, the primary myocardial energy reserve pathway for generating ATP is the creatine kinase reaction (54). Several studies report a significant depletion of creatine, phosphocreatine, creatine kinase levels, and creatine transporter activities in heart tissues (55, 56), and this evidence has led to the hypothesis that the failing heart could be energy-starved (57). Our data show a reduction of the creatine levels in the sera of deceased patients with HF as compared with survivors. We can hypothesize that long-survival of patients with HF better compensates the heart's energetic demand by enhancing creatine synthesis and transport, whereas deceased patients could no longer cope with the myocardium energetic needs, and, thus, the decrease of creatine levels may indicate a state of energy depletion.

Creatine is non-enzymatically converted in creatinine by muscle at an almost constant rate depending on muscle mass, and, then, creatinine is excreted by the kidneys into the urine (53). Elevated levels of serum creatinine are associated with impaired kidney function and renal failure; thus, in clinical practice, it is routinely used as a marker of renal function. In our cohort, we observed higher serum creatinine levels in patients with HF with poor prognosis, despite, at the time of blood sample collection, only 4 (15.4%) out of the 26 deceased patients with HF presented overt chronic renal failure (whereas none of the survived patients with HF showed this comorbidity). The general increment of creatinine in deceased patients, although within the normal range for most of them, could be interpreted as a very early prodromal sign of future renal damage, and it can be associated with poor prognosis. This result is in line with the evidence that a loss of glomerular filtration rate independently predicts mortality and accelerates the overall progression of cardiovascular disease and HF (58). Indeed, the heart and kidneys interact in a complex, bidirectional, and interdependent manner in both acute and chronic settings, by sharing several inflammatory, metabolic, and hormonal pathways (59).

Interestingly, deceased patients with HF showed increased levels of trimethylamine-N-oxide (TMAO), a metabolite generated by gut microbiota from dietary precursors rich in choline, phosphatidylcholine, and l-carnitine. The altered intestinal function has long been associated with HF pathogenesis (60), and a positive correlation between blood levels of TMAO and 5-year risk of death in patients with HF was reported (61). Furthermore, within the cardiovascular setting, the TMAO accumulation has been linked with platelet hyperactivation, atherogenesis, and future adverse cardiac events (i.e., myocardial infarction, stroke, and cardiovascular death) (60).

The ability of the metabolomic fingerprint to distinguish deceased and survived patients with HF is also significantly affected by the levels of several HDL subfractions of apo-A1, apo-A2, cholesterol, and triglycerides. In particular, the patients with HF with poor prognoses were associated with reduced levels of various HDL cholesterol subfractions and VLDL triglyceride subfractions. These data are in agreement with the evidence that low HDL-cholesterol, apo-A1, and triglycerides levels correlate with adverse prognosis in patients with heart failure independent of the etiology (62–64), probably because apo-A1 may exert an anti-inflammatory action in HF (65).

Although none of the just discussed metabolites has sufficient diagnostic power by itself, each of them contributes to the metabolic fingerprint of the patients with HF, and this is the most innovative point of the approach described here. The metabolic fingerprint can be thought of as a sort of holistic super-biomarker with a discriminative power higher than the simple sum of the few quantified metabolites because it takes into account all the detectable signals of endogenous and exogenous metabolites/lipoproteins present in the NMR spectra (66). The metabolomic fingerprint, as a whole, represents, therefore, a useful and innovative instrument that is able to accurately identify the patients with HF with good and poor prognosis, even when compared with more classical stratification approaches. After univariate analysis, metabolomic fingerprint, advanced (>60 years) age at DCM diagnosis, longer (>15 years) history of DCM, NT-proBNP values, and LVEF were significantly related to CV outcomes. With regards to the prognostic power of known risk factors compared with metabolomics results, our data showed higher specificity (71.2%) of NT-proBNP in discriminating deceased from survivor patients, despite lower sensitivity (58.3%) and accuracy (68%). Furthermore, only age, metabolomics, and LVEF have maintained their prognostic significance after multivariate analysis. As previously reported in the literature, younger age is a known protective factor in patients with HF (67, 68) and this finding is confirmed by our results. The importance of LVEF in the prognostic stratification of patients with HF is corroborated as well. In particular, LVEF remains one of the most powerful prognostic factors; indeed, patients with reduced LVEF were at higher risk of death (HRs 7.47 and 4.26, *p*-values 0.0001 and 0.0002 as compared with LR and IR, respectively), whereas patients with mid-range and preserved LVEF shared a lower risk (HR 1.79, *p*-value 0.38). When testing the potential of metabolomics in sub-stratifying LVEF classes, we considered mid-range and preserved LVEF as a unique low-risk class, whereas reduced LVEF was considered as a high-risk class; though metabolomics showed to effectively sub-stratify low and high-risk patients in both classes (HRs 3.46 and 6.01, *p*-values 0.03 and 0.004, respectively), it showed the best performance in the high-risk class. Furthermore, the outcome prediction of the combination of metabolomics and LVEF led to an excellent prognostic power with 75.5% accuracy, 73.8% sensitivity, and 80.8% specificity, (HR 8.09 and *p*-value 0.0000004), highlighting that metabolomic fingerprinting and LVEF provide complementary prognostic information, and, therefore, their combined use can improve poor outcome prediction beyond the use of LVEF only.

In conclusion, the results of this retrospective and proof-of-concept study demonstrate how metabolomic analysis *via* NMR enables a fast and reproducible characterization of the serum metabolic fingerprint associated with poor prognosis in a population of oligosymptomatic patients with HF, improving the cardiovascular risk assessment, and most likely identifying patients with HF who need to undergo more aggressive treatments. Thus, metabolomic fingerprinting could represent a valid addition to the established prognostic instruments, like LVEF. Furthermore, our results suggest that it would be advisable to integrate more risk parameters to identify earlier the patients with HF at high-risk of poor outcomes.

This study provides important insights into the HF setting by analyzing a well-defined, homogeneous, single-center, cohort of patients with DCM with stable chronic HF. As compared to other studies aimed at using metabolomics as a prognostic factor of mortality in patients with HF (31–33), in our study, the patients were monitored for follow up time significantly longer than the average follow ups time of clinical trials (median follow up from enrollment 8 years, median follow up from DCM diagnosis 15 years), most patients were pauci-symptomatic at enrollment (85.8% NYHA class I-II) and 68.8% of patients showed mid-range or preserved LVEF; this sub-group of patients with HF is probably the one that could gain more benefits from specific targeted therapies. However, some relevant limitations of our study should also be mentioned: the population sample was limited in size, the number of death events was low, and the study lacks an independent external validation cohort. These limitations prevent any definitive conclusion. However, the results obtained in this pilot study provide a rational basis for a future larger multi-center study, and further efforts in this direction are guaranteed.

## Data Availability Statement

The original contributions presented in the study are included in the article/Supplementary Materials, further inquiries can be directed to the corresponding authors.

## Ethics Statement

The studies involving human participants were reviewed and approved by Ethics Committee (Azienda Ospedaliero—Universitaria Careggi, Florence, Italy). The patients/participants provided their written informed consent to participate in this study.

## Author Contributions

LT, GC, BA, and CL contributed to study conception and design. AF, GC, IO, NM, and BA contributed to patient enrollment and management. AF, GC, EC, IO, and BA contributed to collection of clinical data and serum samples. AV and LT performed NMR analysis, statistical analysis, biostatistics, and computational analysis. AV, AF, LT, GC, IO, BA, and CL contributed to results interpretation. AV and AF wrote the original draft. AV, AF, LT, GC, IO, and CL wrote, reviewed, and edited the manuscript. NM, BA, and CL contributed to supervision. All authors have read and agreed to the published version of the manuscript.

## Conflict of Interest

The authors declare that the research was conducted in the absence of any commercial or financial relationships that could be construed as a potential conflict of interest.

## Publisher's Note

All claims expressed in this article are solely those of the authors and do not necessarily represent those of their affiliated organizations, or those of the publisher, the editors and the reviewers. Any product that may be evaluated in this article, or claim that may be made by its manufacturer, is not guaranteed or endorsed by the publisher.
