# Supplementary material for: Metabolomics Fingerprint Predicts Risk of Death in Dilated Cardiomyopathy and Heart Failure
Source: Front Cardiovasc Med. 2022 Apr 7;9:851905. doi: 10.3389/fcvm.2022.851905 (PMC9021397; doi:10.3389/fcvm.2022.851905)
Supplement: Supplementary file 1 [file Data_Sheet_1.PDF]

Supplementary Material

1 Supplementary Tables

Supplementary Table S1. Univariate analysis of the 136 metabolomic parameters quantified (22 metabolites, 114 lipoprotein-related parameters) for the comparison between survived and deceased HF patients. The median and MAD in the two groups, the *p*-value of the Wilcoxon Rank-Sum test together with the *p*-value adjusted for false discovery rate with the Benjamini-Hochberg correction (α = 0.05) are reported for each parameter.

Metabolomic parameter	Survived pts		Deceased pts		*p*-value	FDR *p*-value
	Median	MAD	Median	MAD		
Apo A2 HDL (mg/dL)	36.46	5.22	31.27	6.5	0.003	0.26
Apo A2 (mg/dL)	35.84	5.25	31.52	7.06	0.01	0.26
Phospholipids HDL 3 (mg/dL)	17.88	4.92	16.73	3.83	0.01	0.26
Trimethylamine-N-oxide (mmol/L)	0.01	0.01	0.02	0.01	0.01	0.26
Creatinine (mmol/L)	0.1	0.03	0.12	0.04	0.01	0.26
Lactate (mmol/L)	2.25	0.69	2.57	1	0.01	0.26
Apo A1 HDL 3 (mg/dL)	29.2	8.1	25.61	5.79	0.02	0.26
Apo A1 HDL 4 (mg/dL)	80.75	12.42	73.27	17.2	0.02	0.26
Apo A2 HDL 4 (mg/dL)	21.58	5.03	18.89	5.68	0.02	0.26
Creatine (mmol/L)	0.02	0.02	0.01	0.01	0.02	0.26
LDL Chol/HDL Chol	1.84	0.6	2.18	0.67	0.03	0.26
Phospholipids VLDL (mg/dL)	36.39	17.74	28.3	9.03	0.03	0.26
Triglycerides LDL 3 (mg/dL)	2.54	0.81	3.05	0.95	0.03	0.26
Phospholipids HDL 4 (mg/dL)	30.54	5.2	28.87	5.69	0.03	0.26
Apo A2 HDL 3 (mg/dL)	7.46	1.78	6.84	1.85	0.03	0.26
Free Cholesterol VLDL 3 (mg/dL)	2.8	1.86	1.86	1.22	0.04	0.26
Triglycerides LDL 2 (mg/dL)	2.34	0.9	2.77	0.99	0.04	0.26
Triglycerides HDL 4 (mg/dL)	4.54	1.67	3.91	1.42	0.04	0.26
Cholesterol HDL 3 (mg/dL)	9.85	2.91	9.37	3.04	0.04	0.26
Particle Number LDL 2 (nmol/L)	135.41	69.26	162.49	75.8	0.05	0.26
Free Cholesterol VLDL (mg/dL)	15.61	7.15	12.62	4.17	0.05	0.26
Triglycerides VLDL 2 (mg/dL)	24.32	15.16	18.5	10.99	0.05	0.26
Triglycerides VLDL 3 (mg/dL)	19.32	11.22	14.75	8.12	0.05	0.26
Cholesterol VLDL 3 (mg/dL)	5.32	3.51	3.94	2.77	0.05	0.26
Phospholipids VLDL 2 (mg/dL)	6.28	3.77	4.66	2.66	0.05	0.26

Phospholipids VLDL 3 (mg/dL)	5.99	3.14	4.54	2.37	0.05	0.26
Apo B LDL 2 (mg/dL)	7.45	3.82	8.93	4.17	0.05	0.26
Triglycerides VLDL(mg/dL)	146.53	95.28	103.81	64.2	0.06	0.26
Cholesterol VLDL 2 (mg/dL)	6	4.06	3.67	2.54	0.06	0.26
Free Cholesterol VLDL 2 (mg/dL)	2.49	1.67	1.73	1.19	0.06	0.26
Triglycerides HDL 3 (mg/dL)	2.8	1.19	2.37	0.77	0.06	0.26
Cholesterol HDL 4 (mg/dL)	20.17	4.06	18.83	5.32	0.06	0.26
Acetate (mmol/L)	0.02	0.01	0.02	0.01	0.06	0.26
Cholesterol VLDL (mg/dL)	33.79	19.37	26.16	10.3	0.07	0.29
Triglycerides LDL 6 (mg/dL)	5.64	2.28	4.72	1.92	0.07	0.29
Free Cholesterol VLDL 5 (mg/dL)	0.88	0.62	0.68	0.38	0.08	0.29
Phospholipids VLDL 1 (mg/dL)	12.66	10.04	7.32	4.39	0.08	0.29
Cholesterol LDL 2 (mg/dL)	12.56	7.23	14.5	8.7	0.08	0.29
Phospholipids LDL 2 (mg/dL)	7.29	3.44	8.11	4.54	0.08	0.29
Apo A1 (mg/dL)	150.13	23.96	141.72	26.28	0.09	0.31
Phospholipids HDL (mg/dL)	79.25	17.72	74.61	14.86	0.09	0.31
Triglycerides VLDL 1 (mg/dL)	78.7	64.66	46.82	28.05	0.09	0.31
Triglycerides IDL (mg/dL)	27.13	23.06	16.69	15.37	0.1	0.31
Phospholipids IDL (mg/dL)	11.45	6.3	9.04	3.25	0.1	0.32
Cholesterol VLDL 1 (mg/dL)	13.95	10.4	9.14	6.54	0.1	0.31
Free Cholesterol LDL (mg/dL)	32.59	9.85	34.17	10.44	0.11	0.32
Apo A1 HDL (mg/dL)	149.97	22.67	144.97	32.38	0.11	0.32
Phospholipids LDL 6 (mg/dL)	16.02	5.92	13.05	3.91	0.11	0.32
Particle Number LDL 3 (nmol/L)	135.19	98.76	179.03	87.34	0.12	0.33
Free Cholesterol VLDL 1 (mg/dL)	5.58	4.65	3.4	2.5	0.12	0.33
Apo B LDL 3 (mg/dL)	7.44	5.43	9.84	4.8	0.12	0.33
Glutamine (mmol/L)	0.73	0.09	0.78	0.11	0.12	0.33
Triglycerides (mg/dL)	213.77	126.1	154.68	93.49	0.13	0.33
Cholesterol LDL 3 (mg/dL)	11.39	9.85	15.41	10.02	0.13	0.33
Phospholipids LDL 3 (mg/dL)	6.88	5.29	8.86	4.77	0.13	0.33
Apo A1 HDL 2 (mg/dL)	18.98	4.74	17.95	4.18	0.13	0.33
Triglycerides VLDL 4 (mg/dL)	10.75	5.19	9.91	3.91	0.14	0.33
Glucose (mmol/L)	5.22	1.05	5.31	0.95	0.14	0.33
Particle Number VLDL (nmol/L)	221.54	113.99	182.77	49.9	0.15	0.33
Apo B VLDL (mg/dL)	12.18	6.27	10.05	2.74	0.15	0.33
Triglycerides HDL 2 (mg/dL)	2.25	0.96	2.05	0.53	0.15	0.33
Cholesterol LDL 6 (mg/dL)	27.67	10.89	23.44	8.19	0.16	0.34
Free Cholesterol LDL 2 (mg/dL)	4.43	2.13	4.88	2.5	0.16	0.34

Apo B100/Apo A1	0.62	0.17	0.68	0.22	0.17	0.35
Particle Number LDL 6 (nmol/L)	442.9	187.18	361.11	139.65	0.17	0.34
Triglycerides HDL (mg/dL)	13.02	4.92	12.21	3.63	0.17	0.34
Apo B LDL 6 (mg/dL)	24.36	10.29	19.86	7.68	0.17	0.34
Dimethylsulfone (mmol/L)	0.01	0	0.01	0.01	0.18	0.36
Triglycerides VLDL 5 (mg/dL)	3.09	0.87	3.06	0.68	0.19	0.37
Cholesterol LDL 1 (mg/dL)	20.85	5.92	21.91	7.11	0.19	0.37
Apo A2 HDL 2 (mg/dL)	3.88	1.2	3.59	1.17	0.19	0.37
Triglycerides LDL 4 (mg/dL)	2.57	1.21	3.02	1.3	0.2	0.37
Free Cholesterol LDL 3 (mg/dL)	4.13	2.67	4.48	2.53	0.2	0.37
Phospholipids HDL 2 (mg/dL)	13.28	3.48	11.58	3.62	0.2	0.37
Free Cholesterol LDL 1 (mg/dL)	6.27	2.11	6.75	1.79	0.22	0.39
Free Cholesterol HDL 4 (mg/dL)	4.12	1.36	3.7	1.41	0.22	0.39
Citrate (mmol/L)	0.14	0.03	0.16	0.04	0.23	0.41
Free Cholesterol HDL 3 (mg/dL)	2.54	1.02	2.46	0.9	0.24	0.42
Acetone (mmol/L)	0.03	0.01	0.04	0.02	0.24	0.42
LDL Cholesterol (mg/dL)	113.89	33.23	106.78	33.38	0.25	0.42
Cholesterol LDL (mg/dL)	113.89	33.23	106.78	33.38	0.25	0.42
Free Cholesterol VLDL 4 (mg/dL)	2.3	1.48	2.12	1.25	0.25	0.42
Particle Number LDL 1 (nmol/L)	221.58	51.36	224.25	76.05	0.26	0.42
Free Cholesterol LDL 6 (mg/dL)	7.34	2.6	6.56	2.48	0.26	0.42
Free Cholesterol HDL 1 (mg/dL)	3.92	1.99	4.56	1.29	0.26	0.42
HDL Cholesterol (mg/dL)	55.03	12.82	55.25	13.61	0.27	0.42
Cholesterol HDL (mg/dL)	55.03	12.82	55.25	13.61	0.27	0.42
Apo B LDL 1 (mg/dL)	12.18	2.83	12.33	4.17	0.27	0.42
Phospholipids LDL 1 (mg/dL)	12.55	2.91	13.02	3.61	0.28	0.42
Cholesterol IDL (mg/dL)	16.18	8.99	14.17	7.2	0.29	0.42
Phospholipids VLDL 4 (mg/dL)	5.37	2.03	4.84	1.93	0.29	0.43
Phospholipids VLDL 5 (mg/dL)	1.92	0.91	1.82	0.68	0.29	0.42
Cholesterol HDL 2 (mg/dL)	7.86	2.61	6.96	2.66	0.29	0.42
Phospholipids LDL (mg/dL)	64.16	18.63	63.05	16.74	0.31	0.45
Free Cholesterol IDL (mg/dL)	4.87	2.56	3.98	1.93	0.33	0.47
Alanine (mmol/L)	0.47	0.09	0.5	0.09	0.36	0.51
Cholesterol VLDL 4 (mg/dL)	5.96	3.18	5.38	2.9	0.38	0.53
Apo A1 HDL 1 (mg/dL)	23.89	9.51	25.07	8.83	0.39	0.54
Cholesterol LDL 4 (mg/dL)	13.48	11.63	13.87	9.09	0.4	0.54
Particle Number LDL 4 (nmol/L)	164.23	125.63	183.25	99.83	0.41	0.55
Phospholipids LDL 4 (mg/dL)	7.87	6.3	7.53	4.26	0.41	0.55
Apo B LDL 4 (mg/dL)	9.04	6.91	10.08	5.49	0.41	0.55

Glycine (mmol/L)	0.25	0.08	0.22	0.07	0.41	0.55
Cholesterol VLDL 5 (mg/dL)	1.31	0.83	1.36	0.62	0.45	0.59
Cholesterol (mg/dL)	218.12	41.24	205.64	40.36	0.46	0.59
Triglycerides LDL 1 (mg/dL)	6.71	2.77	7.71	2.33	0.46	0.59
Free Cholesterol LDL 4 (mg/dL)	4.02	2.68	4.43	2.77	0.46	0.59
Tyrosine (mmol/L)	0.07	0.02	0.08	0.01	0.48	0.6
Particle Number LDL (nmol/L)	1349.74	436.83	1394.18	329.94	0.5	0.62
Apo B LDL (mg/dL)	74.23	24.03	76.68	18.15	0.5	0.62
Free Cholesterol HDL (mg/dL)	12.73	4.66	12.89	3.75	0.51	0.62
Leucine (mmol/L)	0.13	0.04	0.12	0.04	0.51	0.62
Triglycerides HDL 1 (mg/dL)	3.92	2.01	3.7	1.43	0.54	0.66
Triglycerides LDL (mg/dL)	23.15	7.64	23.21	4.39	0.65	0.77
Isoleucine (mmol/L)	0.07	0.03	0.07	0.03	0.65	0.77
Cholesterol HDL 1 (mg/dL)	15.76	6.28	15.62	5.32	0.66	0.78
Methionine (mmol/L)	0.08	0.02	0.08	0.01	0.69	0.8
Formate (mmol/L)	0.02	0	0.02	0.01	0.69	0.8
Free Cholesterol HDL 2 (mg/dL)	2.01	0.93	2.04	0.88	0.71	0.81
Valine (mmol/L)	0.27	0.05	0.27	0.06	0.77	0.88
Acetoacetate (mmol/L)	0.01	0	0.01	0	0.79	0.89
Phenylalanine (mmol/L)	0.07	0.02	0.07	0.02	0.8	0.89
Particle Number IDL (nmol/L)	121.87	49.93	128.22	46.72	0.81	0.9
Apo B IDL (mg/dL)	6.7	2.74	7.05	2.56	0.83	0.9
Triglycerides LDL 5 (mg/dL)	3.38	1.48	3.1	0.99	0.83	0.9
Apo A2 HDL 1 (mg/dL)	2.41	1.07	2.54	0.72	0.84	0.9
Cholesterol LDL 5 (mg/dL)	19	10.39	18.55	7.77	0.85	0.9
Histidine (mmol/L)	0.1	0.02	0.09	0.01	0.85	0.9
Phospholipids HDL 1 (mg/dL)	18.35	6.35	17.68	6.63	0.86	0.9
Pyruvate (mmol/L)	0.07	0.03	0.06	0.05	0.88	0.92
Free Cholesterol LDL 5 (mg/dL)	5.18	2.44	5.42	2.38	0.9	0.93
Phospholipids LDL 5 (mg/dL)	10.59	5.09	10.33	3.5	0.9	0.93
Apo B100 (mg/dL)	96.87	22.72	92.65	24.69	0.93	0.94
Total Particle Number (nmol/L)	1761.38	413.13	1684.62	448.89	0.93	0.94
Particle Number LDL 5 (nmol/L)	245.59	126.2	248.25	108.36	0.99	0.99
Apo B LDL 5 (mg/dL)	13.51	6.95	13.65	5.96	0.99	0.99

2 Supplementary Figures

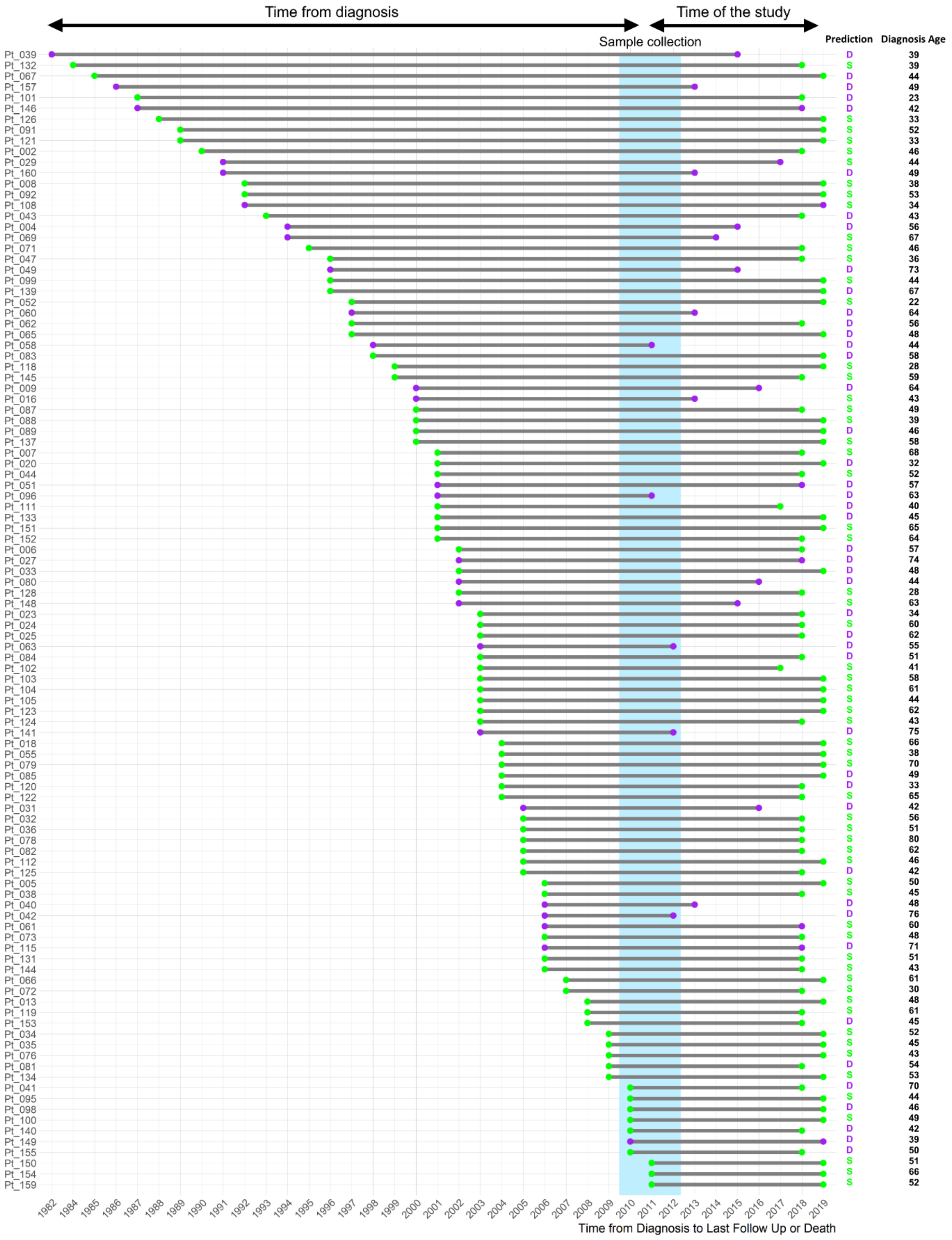

Supplementary Figure S1. Segment plot reporting for each patient the time of diagnosis and last follow up/death. Green dots: survived patients, purple dots: deceased patients. The metabolomics prediction and the age are also reported.

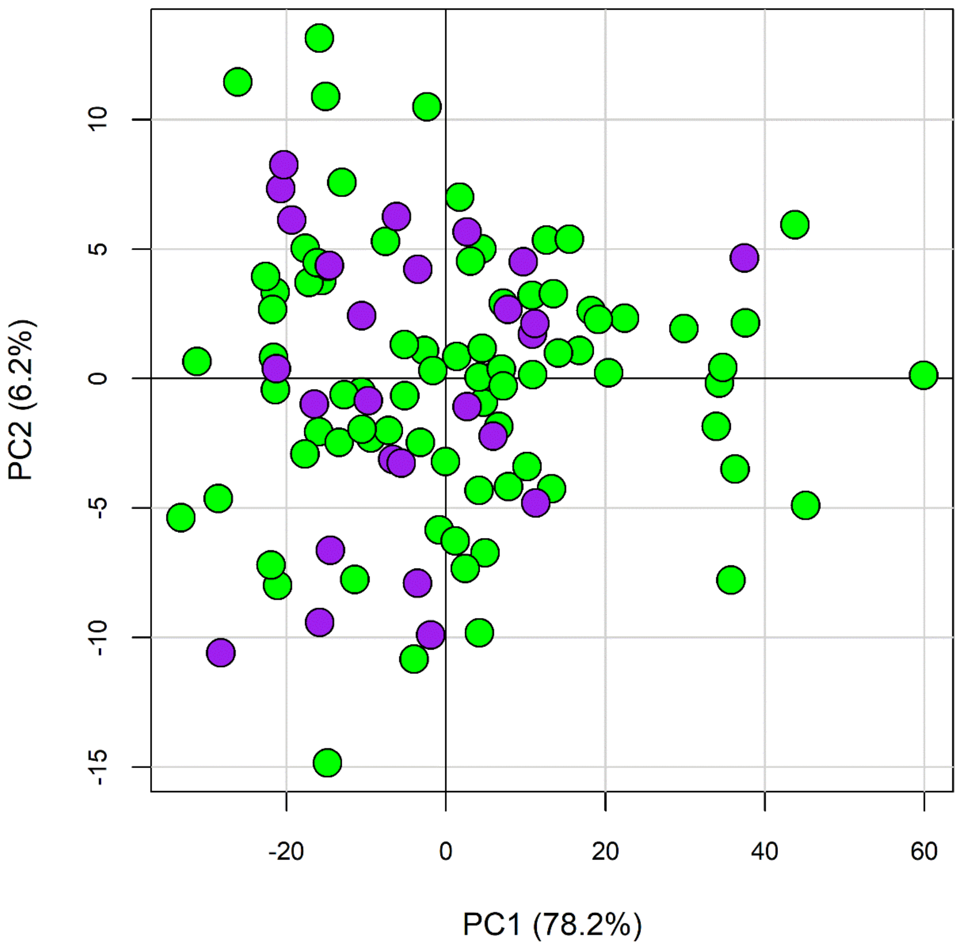

Supplementary Figure S2. Score plot of the first two principal components of the PCA model. Circles are colored according to the survival status: survivor (80) in green and deceased (26) in purple. For each PC, the percentage of explained variance is also reported.

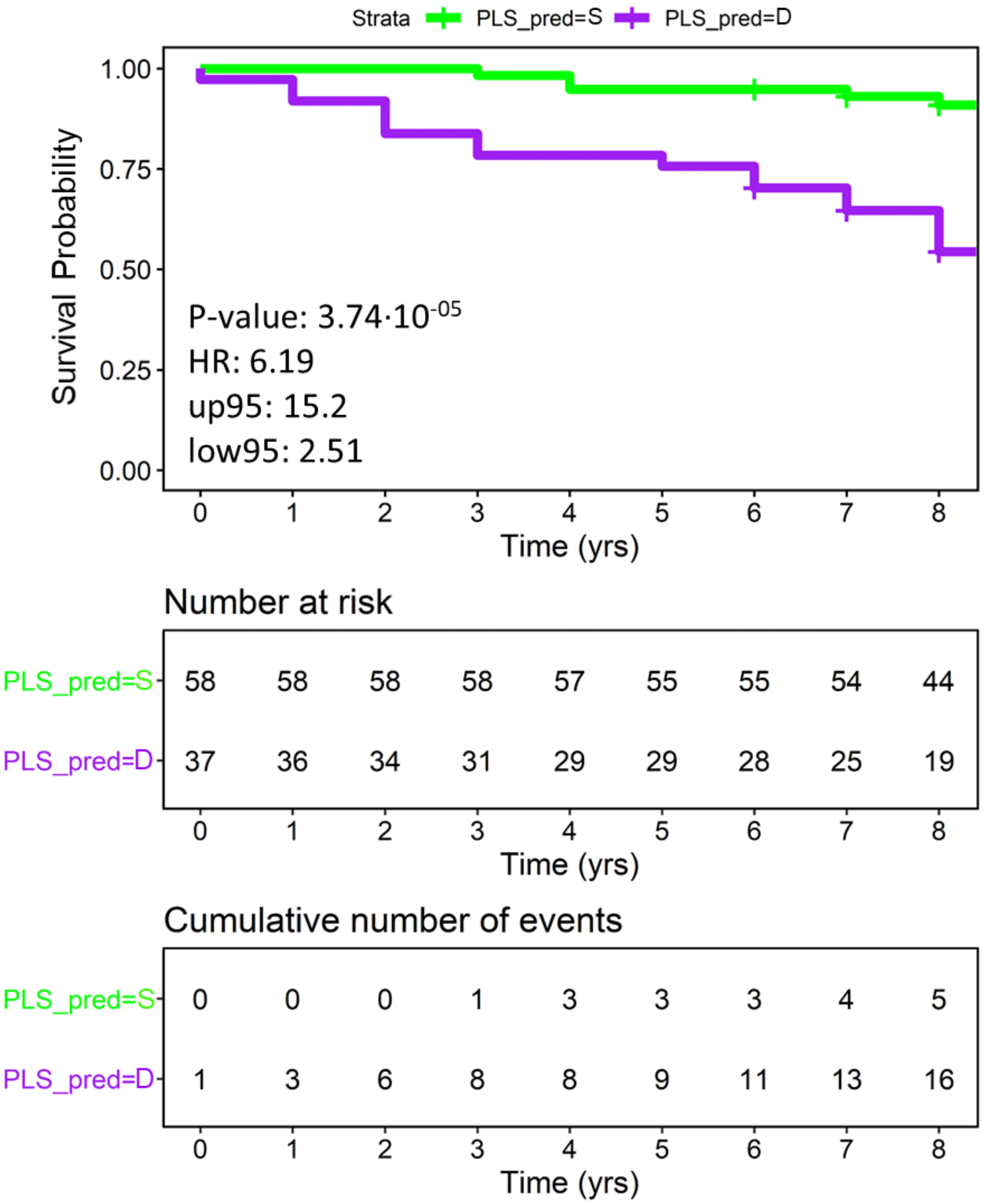

Supplementary Figure S3. HF patient analysis removing 11 borderline subjects, plotting actual survival over time (measured in years) according to estimated risk based on metabolomics only or on the combined score (Kaplan-Meier curves). Low risk patients are colored in green and high risk in purple. P-value are calculated using the Log-Rank test. Censored events represent either the time of last recorded clinical follow up, or time of death. Number at risk: number of patients stratified according to metabolomic score at each time point. Cumulative number of events: total number of deceased patients at each time point for each risk group based on metabolomics score. Kaplan-Meier analysis according to metabolomics risk estimation.

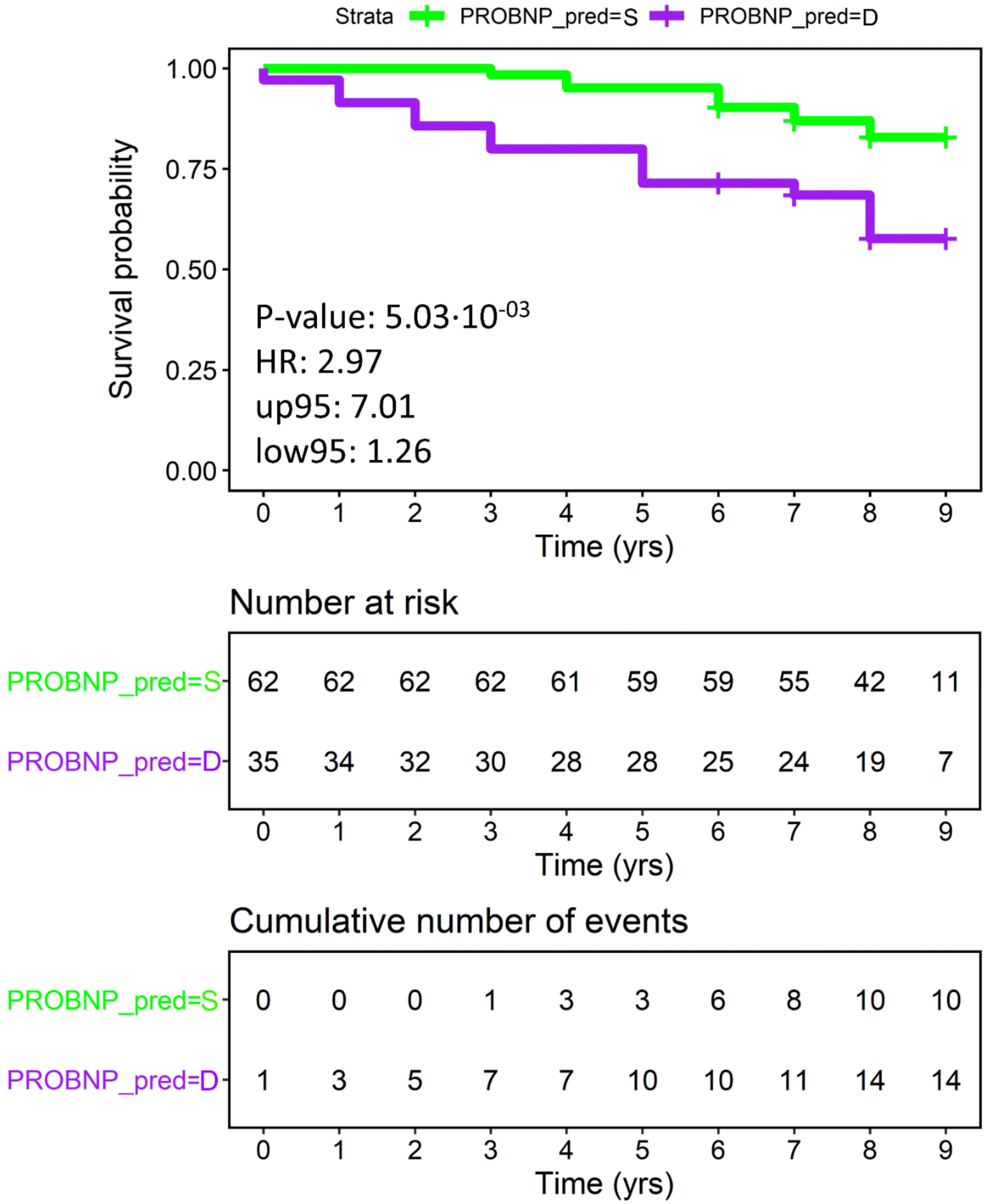

Supplementary Figure S4. Overall HF patients, plotting actual survival over time (measured in years) according to estimated risk based on NT-proBNP (Kaplan-Meier curves). Low risk patients are colored in green and high risk in purple. P-value are calculated using the Log-Rank test. Censored events represent either the time of last recorded clinical follow up, or time of death. Number at risk: number of patients stratified according to NT-proBNP. Cumulative number of events: total number of deceased patients at each time point for each NT-proBNP group. Kaplan-Meier analysis on NT-proBNP, patients with NT-proBNP > 400 pg/mL were considered at high risk of death.

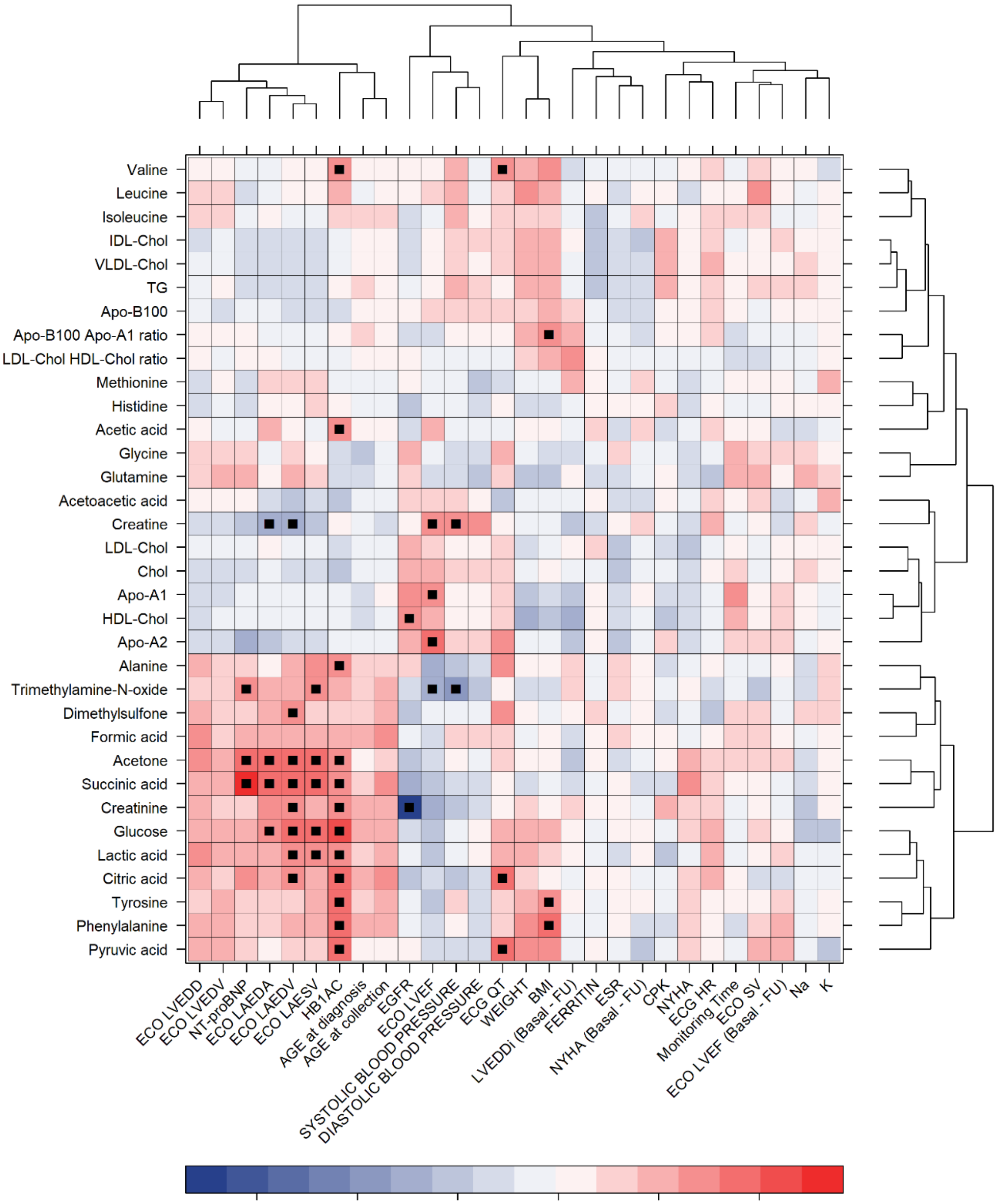

Supplementary Figure S5. Heatmap hierarchically clustered showing correlations between clinical variables and metabolic parameters (metabolites and main lipoprotein-related parameters). R values are shown as different degree of color intensity (red, positive correlations; blue, negative correlation). ■ statistically significant correlations (p-value < 0.05 after FDR correction). Abbreviations: ESR: Erythrocyte sedimentation rate; CPK: creatine phosphokinase; EGFR: Estimated glomerular filtration rate; HB1AC: Glycated hemoglobin; ECG HR: ECG heart rate; ECO LAEDV: ECO left atrial end diastolic volume; ECO LAEDA: ECO left atrial end diastolic area; ECO LAESV: ECO left atrial end systolic volume; ECO LVEDD: ECO left ventricular end diastolic diameter; ECO LVEDV: ECO left ventricular end diastolic volume; ECO SV: ECO stroke volume; ECO LVEF: ECO ejection fraction; FU: last follow up; NYHA: New York Heart Association classification.

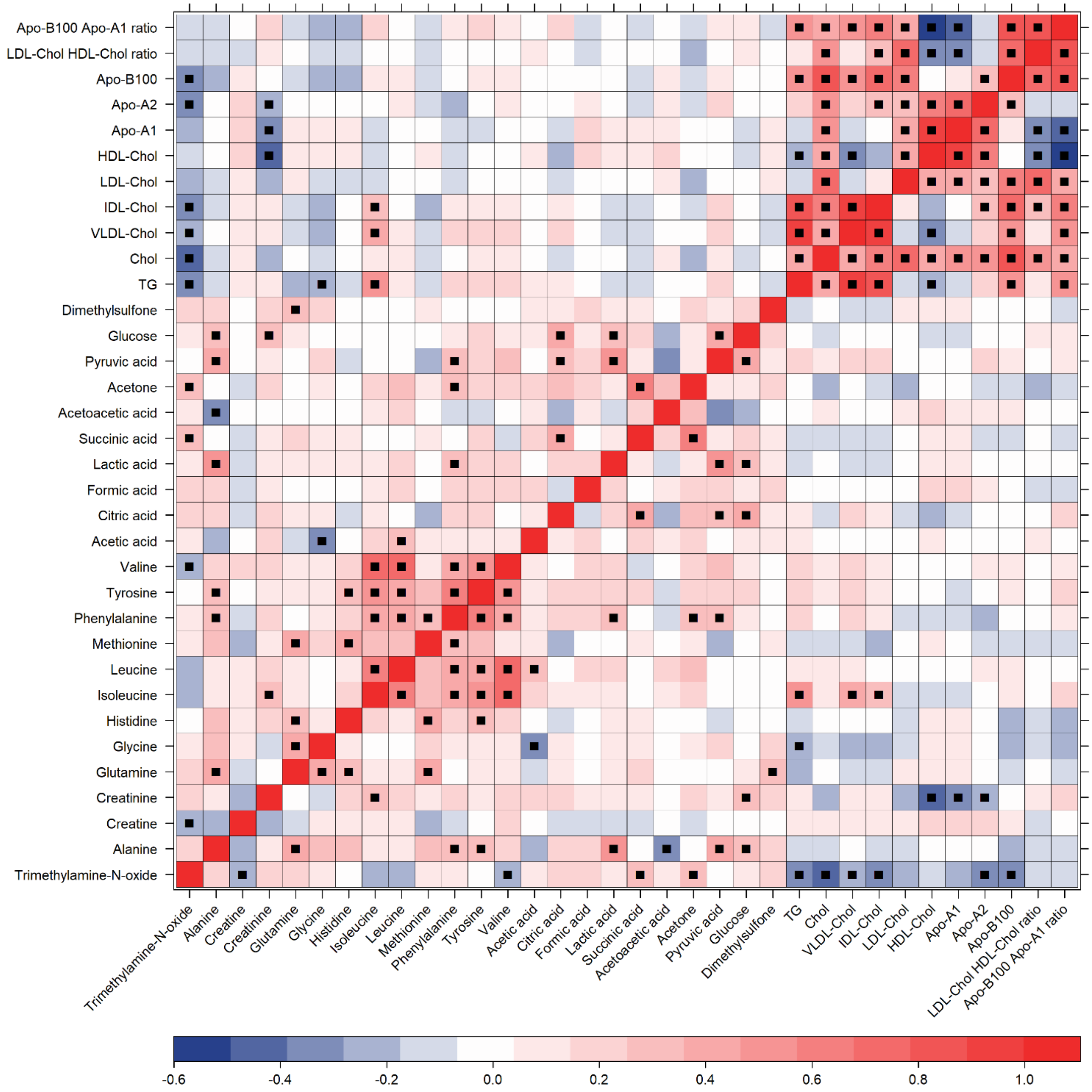

Supplementary Figure S6. Heatmap hierarchically clustered showing correlations among metabolic parameters (metabolites and main lipoprotein-related parameters). R values are shown as different degree of color intensity (red, positive correlations; blue, negative correlation). ■ statistically significant correlations (p-value < 0.05 after FDR correction).
